# Comprehensive Characterization of the Oxidative Stress Profiles in Neonatal Necrotizing Enterocolitis

**DOI:** 10.7150/ijms.109008

**Published:** 2025-04-09

**Authors:** Xiaofeng Xiong, Luyao Wu, Xin Liu, Jing Wang, Jun Xiao, Ke Chen, Didi Zhuansun, Xinyao Meng, Jiexiong Feng, Xuyong Chen

**Affiliations:** 1 Department of Pediatric Surgery, Tongji Hospital, Tongji Medical College, Huazhong University of Science and Technology, Wuhan, China.; 2 Hubei Clinical Center of Hirschsprung disease and allied disorders, Wuhan, China.; 3 Department of Operating Room, Tongji Hospital, Tongji Medical College, Huazhong University of Science and Technology, Wuhan, China.

**Keywords:** oxidative stress, neonatal necrotizing enterocolitis, immune infiltration, macrophages, single cell RNA-sequencing.

## Abstract

**Objective:** This study aims to portray the characteristics of oxidative stress (OS) in cases of Necrotizing enterocolitis (NEC), identify the hub genes and associated mechanisms involved, and explore potential drugs for NEC.

**Methods:** We performed a comprehensive analysis integrating bulk-RNA sequencing and single-cell RNA sequencing datasets, coupled with various techniques including differential analysis, gene set enrichment analysis, and immune infiltration analysis. We aimed to systematically elucidate the variations in functions related to OS among distinct cell populations within both NEC and non-NEC tissues. Additionally, we depicted the longitudinal changes in immune cells, with a particular focus on macrophages, throughout the progression of NEC. NEC mice model was established and RT-qPCR was performed to validate the expression of the hub genes.

**Results:** In total, 465 OS related genes were found, and 53 of them were significantly differentially expressed. These genes were mainly involved in several signaling pathways, such as TNF signaling pathway, IL-17 signaling pathway, FOXO signaling pathway, inflammatory bowel disease. The top 10 hub genes were *MMP2*, *IL1A*, *MMP3*, *HGF*, *HP*, *IL10*, *PPARGC1A*, *TLR4*, *MMP9* and *HMOX1*. Ten kinds of drug were discovered as the potential treatment for NEC. Four specific macrophages subtypes and relative function were identified in NEC. RT-qPCR and immunofluorescence staining confirmed the expression of the hub genes in NEC model.

**Conclusions:** This investigation yielded innovative insights into the immune environment and therapeutic methodologies directed at oxidative stress in the pathogenesis of NEC.

## Introduction

Necrotizing enterocolitis (NEC) stands as a pivotal contributor to morbidity and mortality in premature infants. Impacting 1-3 cases per 1000 live births, this gastrointestinal ailment predominantly targets prematurely born infants. The risk is notably inversely associated with both gestational age and birth weight 1. Despite advancements in perinatal and neonatal care, NEC remains a substantial contributor to morbidity and mortality among preterm infants. The mortality rates associated with NEC are estimated to be around 30% 2. NEC is a complex condition characterized by a pathogenesis that is not fully understood. Prematurity is acknowledged as the foremost risk factor, and factors such as intestinal immaturity, dysregulated microvascular tone, aberrant microbial colonization in the intestine, dysregulated immune response, and hypoxic-ischemic injury collectively contribute to the onset of intestinal inflammation and necrosis 3. Given the multifaceted nature of NEC and its frequently abrupt onset, there remains ongoing debate regarding its definitive treatment and management.

Newborns, especially those born prematurely, exhibit heightened susceptibility to oxidative stress (OS) in comparison to children and adults. This vulnerability stems from an elevated production of reactive oxygen species (ROS), capable of inflicting damage on various organs and tissues in neonates 4. Extensive research has been dedicated to investigating the involvement of ROS in the pathogenesis of various neonatal diseases. In 1988, Saugstad introduced the term "oxygen radical diseases of neonatology" to encompass conditions such as respiratory distress syndrome, bronchopulmonary dysplasia, periventricular leukomalacia, patent ductus arteriosus, retinopathy of prematurity, and NEC. Various processes, encompassing hypoxia, ischemia-reperfusion, hyperoxia, activation of neutrophils and macrophages, mitochondrial dysfunction, endothelial cell injury, and prostaglandin metabolism, contribute to the generation of ROS. Recent research has additionally underscored the close relationship between ROS and antioxidant capacity in the pathophysiology of NEC 5.

In the genomics era, high-throughput sequencing is commonly employed to investigate the mechanisms of diseases, offering novel perspectives into the genetic underpinnings of pathogenesis. This study aims to scrutinize the landscape of oxidative stress in NEC through bioinformatics analysis, and discover potential preventive and therapeutic antioxidant strategies for NEC.

## Materials and Methods

### Data source

To obtain gene expression profiles for the study, the datasets included in this study were downloaded from the Gene Expression Omnibus (GEO) database (http://www.ncbi.nlm.nih.gov/geo). The GSE46619 dataset included 5 samples of NEC and 4 samples of spontaneous intestinal perforation for comparison. The GSE64801 dataset contained 9 NEC patients and 5 healthy control patients was used for external validation. The GSE178088 dataset was obtain for single cell RNA-sequencing analysis which contains 2 fetal, 2 neonatal and 2 NEC patients. Specific clinical information of these samples was shown in supplementary table 1.

### Data preprocessing and integration

Differentially expressed genes (DEGs) associated with NEC were identified utilizing the limma package in R, employing an absolute log2 fold change (logFC) greater than 1 and an adjusted P-value less than 0.05 as the criteria for statistical significance. Heat maps and volcano plots depicting the DEGs were generated using the ggplot package in R. To identify genes associated with stress oxidative, we conducted searches in the Gene Ontology (GO) database using both biological process and molecular function criteria. For biological processes, we focused on terms directly related to oxidative stress response pathways, such as "response to oxidative stress" "oxidative stress-induced apoptosis" and "regulation of reactive oxygen species (ROS) metabolic process". Additionally, we included molecular functions known to play key roles in oxidative stress, including "antioxidant activity", "peroxidase activity" and "superoxide dismutase activity" among other enzyme activities involved in ROS regulation and detoxification. By combining the results from these searches, we compiled a comprehensive list of 465 genes strongly associated with oxidative stress, covering a wide range of relevant biological mechanisms. The list of 465 OS related genes was shown in supplementary table 2. The overlap between these genes and the DEGs from the GSE46619 dataset was designated as OS related DEGs.

### Gene set enrichment analysis

In order to attain a more comprehensive understanding of the biological mechanisms inherent to NEC, we performed a Gene Set Enrichment Analysis (GSEA) utilizing the GseaVis and clusterProfiler R packages. We set the cutoff criteria for statistical significance at a false discovery rate (FDR) of less than 0.25 and a P-value of less than 0.05. By applying these criteria, we aimed to identify molecular pathways that were most strongly associated with OS related genes.

### Protein-protein interaction network analysis

The STRING online search tool (http://string-db.org) was utilized to explore interactions between OS related DEGs 6. In order to create a protein-protein interaction (PPI) network with several regulatory links, the tool enables the search for relationships between proteins of interest, including direct binding associations or coexisting upstream and downstream regulatory pathways. A combined score of more than 0.4 was used to classify interactions as statistically significant. Using the Cytoscape program (http://www.cytoscape.org), the PPI network was visualized 7.

### Enrichment analyses of differentially expressed genes

An established international classification system for gene function, the GO offers a carefully defined vocabulary and concepts that are updated constantly to enable thorough identification of genes and their products 8. On the other hand, the Kyoto Encyclopedia of Genes and Genomes (KEGG) serves as the principal public database pertaining to pathways 9. Both GO and KEGG enrichment analyses were performed using the "clusterProfiler" R package or website tool g:Profiler (https://biit.cs.ut.ee/gprofiler/convert).

### Identification and analysis of hub genes

The cytoHubba plug-in of Cytoscape was utilized to identify hub genes. Eleven conventional algorithms, including MNC, EPC, Stress, Radiality, Closeness, Degree, MCC, BottleNeck, EcCentricity, Betweeness, and ClusteringCofficient, were used to assess these hub genes. An upset diagram was employed to filter those essential hub genes that were frequently encountered to all of these methods. Then, a co-expression network comprising these hub genes was created through GeneMANIA (http://www.genemania.org/) 10, which is an established method for uncovering internal associations among gene sets.

### Identification of candidate drugs

The identification of drug molecules is a crucial aspect of the ongoing research on the prevention and treatment of NEC. In order to design drug molecules, DSigDB database is obtained via the Enrichr platform (https://amp.pharm.mssm.edu/Enrichr/), which is mostly utilized for enrichment analysis and provides extensive illustration of functional information corresponding to the input genes 11.

### Immune cell infiltration analysis

The CIBERSORT deconvolution algorithm, accessible at https://cibersortx.stanford.edu/, was employed to assess variations in immune cell infiltration between NEC and CON samples. CIBERSORT serves as an analytical tool utilizing gene expression data to estimate the proportions of individual cell types within a heterogeneous cell population 12. The results obtained by CIBERSORT were visualized using the corplot, vioplot, and ggplot2 packages in R. Subsequently, we conducted a correlation analysis employing Spearman's rank correlation test to assess the relationships between the 22 immune cell types and the Hub genes.

### Single-cell RNA data integration and processing

The raw scRNA-seq data of NEC and un-NEC related disease bowel tissues were obtained (based on GSE178088). Upon reading and presentation of the entire dataset in R, a stringent filtering procedure was applied. Seurat objects were generated from the scRNA-seq data for each tissue utilizing the "Seurat" package in R (version 4.1.0). The quality control of single cell samples was conducted based on the following criteria: (1) each cell expresses more than 200 genes; (2) the same gene is expressed in a minimum of three cells; (3) percentage of hemoglobin genes < 0.1; (4) mitochondrial UMI counts rate exceeding 20%. Subsequently, the Harmony algorithm was employed to harmonize the expression matrices of individual tissues, addressing batch effects across different tissues. The integrated matrix underwent cell normalization and scaling. In summary, a focused analysis was performed on the top 2,000 highly variable features after normalization, and these features were selected for downstream analysis. Following that, principal component analysis (PCA) was executed using the "RunPCA" function on the scaled data, focusing on the top 2,000 highly variable genes. Subsequently, the top 30 principal components were selected for further application in uniform manifold approximation and projection (UMAP) and t-distributed stochastic neighbor embedding (tSNE) dimensional reduction. Unsupervised cell clustering was accomplished by utilizing the "FindClusters" and "FindNeighbors" functions, relying on the top 30 PCA principles. This approach facilitated the grouping of similar cell populations together, with the resolution parameter set to 0.3. Marker genes for each cell cluster were identified using the "FindAllMarkers" function, employing the criteria of min.pct > 0.1, logfc.threshold > 0.25, and a p-value < 0.01. Immune cells were subsequently isolated and subjected to further subdivision, followed by dimensional reduction through re-tSNE and re-UMAP. Cell types were annotated using singleR and the PanglaoDB database, and the cell count for each type was quantified 13, 14. OS score was calculated in different types of cell population based on the average expression of OS related genes in NEC and CON group.

### The experimental NEC protocol

The experimental NEC model was established as described previously 15. This study complied with the Declaration of Helsinki and was approved by the Review Board of the Ethics Committee of Tongji Hospital. The Institutional Review Board of Tongji Hospital, Tongji Medical College, Huazhong University of Science and Technology approved the protocol of the study. In brief, the one-day-old pups were subjected to hypoxia (5% O_2_, 95% N_2_) for 5 min in a hypoxic chamber and hypothermia (24 ^◦^C) for 5 min twice daily for 4 days as well as formula gavage very 3 h for up to 4 days. The experimental pups were sacrificed on Days 5 using CO_2_ and subjected to various analyses. A short segment of terminal ileum was subjected to tissue section. The other part of terminal ileum was also collected and stored in liquid nitrogen for real-time quantitative PCR (RT-qPCR).

### Hematoxylin and eosin (H&E) staining

H&E staining was performed to assess the degree of intestinal lesions. All intestinal samples obtained were fixed with 4% paraformaldehyde for 24 h, sliced up after paraffin embedding, stained with HE and then examined using an Olympus light microscope.

### Real-time quantitative PCR

RT-qPCR was performed to analyze the mRNA expression of hub genes in NEC samples and normal controls. Total RNA was isolated from intestinal tissues using TRIzol reagent (Life Technologies, Carlsbad, CA) following a standardized protocol. Briefly, tissues were homogenized in 1 ml of TRIzol reagent and incubated at room temperature for 5 minutes to allow complete dissociation of nucleoprotein complexes. Subsequently, 0.2 ml of chloroform was added, and the mixture was vigorously shaken for 15 seconds before being centrifuged at 12,000 rpm (approximately 16,000 × g) for 15 minutes at 4°C. The aqueous phase, containing the RNA, was carefully transferred to a new tube, and 0.5 ml of isopropyl alcohol was added to precipitate the RNA. The mixture was left at room temperature for 10 minutes and then centrifuged at 12,000 rpm (approximately 16,000 × g) for 10 minutes at 4°C. The RNA pellet was washed twice with 75% ethanol, dried at room temperature for about 10 minutes, and finally dissolved in 50 µl of RNase-free water.

The isolated RNA was reverse-transcribed into cDNA using a reverse transcription kit (Vazyme) according to the manufacturer's instructions. The reverse transcription reaction was performed in a total volume of 20 µl, containing 500 ng of total RNA, 1 µl of reverse transcriptase, 4 µl of 5× reaction buffer, and 1 µl of random primers. The reaction was incubated at 37°C for 15 minutes, followed by 85°C for 5 seconds to inactivate the reverse transcriptase enzyme.

Gene expression analysis was performed using RT-qPCR with SYBR Green Master Mix (Vazyme). The RT-qPCR reactions were conducted in a total volume of 20 µL, containing 2 µl of cDNA template, 10 µl of SYBR Green Master Mix, and 0.4 µM of each primer. The thermal cycling conditions were as follows: initial denaturation at 95°C for 3 minutes, followed by 40 cycles of 95°C for 10 seconds, 60°C for 30 seconds, and 72°C for 30 seconds. A final melting curve analysis was performed to confirm the specificity of the amplification products. The relative expression levels of target genes were calculated using the 2^-ΔΔCt method, with β-actin serving as the internal control.

### Immunofluorescence staining and confocal microscopy

Colon tissue sections of experimental NEC model and WT group were performed with immunofluorescence staining using the anti-MMP9 antibody (A0289, ABclonal, Woburn, MA, USA) as previously described 36. All sections were independently evaluated by two researchers with LSM 800 confocal microscope (Carl Zeiss MicroImaging GmbH, Jena, Germany).

### Statistical analysis

Data were analyzed using the GraphPad Prism software and presented as the mean ± SD. Differences between the two groups were analyzed using an unpaired t-test with Welch's correction. p < 0.05 was considered statistically significant. DEGs were visualized using ggplot2 (v3.4.4) for heatmaps and volcano plots. GSEA was performed with GseaVis and clusterProfiler (v4.14.4) to identify pathways enriched in oxidative stress-related genes. The same packages were used for GO and KEGG enrichment analyses. PPI networks were analyzed using the STRING database and visualized with Cytoscape (v3.10.0), and hub genes were identified using the cytoHubba plugin (v0.1.0). Single-cell RNA-seq data were preprocessed, quality-controlled, and clustered with Seurat (v4.1.0). Batch effects were corrected using Harmony (v1.0.0), and marker genes were identified with FindAllMarkers. Correlation analysis was conducted using Spearman's rank correlation test with corplot (v0.84), vioplot (v0.3.0), and ggplot2 (v3.4.4).

## Results

### Identification of differentially expressed OS-related genes in NEC

**Figure 1:**
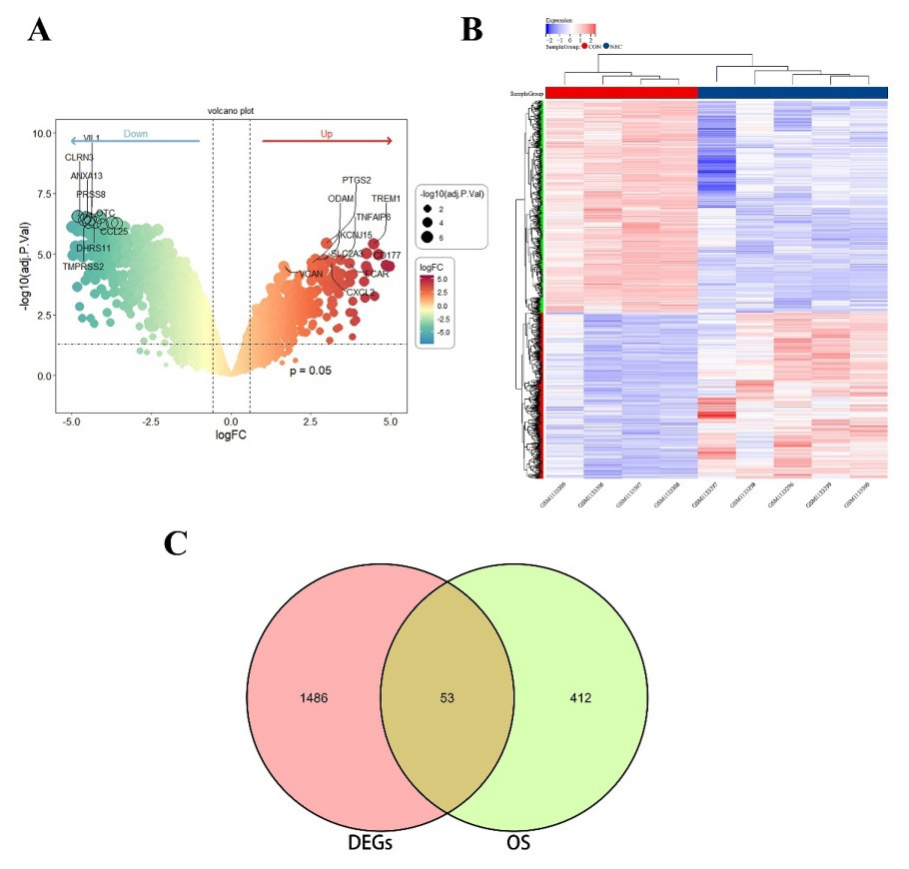
(A) The volcano map of differentially expressed genes (DEGs) in GSE46619. (B) The heatmap and cluster analysis of the gene expression in NEC and CON group. (C) The Venn plot of DEGs and OS related genes showed an overlap of 53 OS related DEGs.

The research flowchart of this study is shown in **Figure S1**. After analysis of the data set GSE46619, a total of 1539 DEGs were found. A set of 465 genes associated with oxidative stress was curated from the GO database. The Venn diagram illustrated an overlap of 53 genes between DEGs and OS related genes, comprising 22 upregulated and 31 downregulated genes. **Figure 1A-B** displays the volcano plot of GSE46619 and the heatmap of the 53 OS related DEGs. The Venn diagram is depicted in **Figure 1C**.

The KEGG gene set database was used to analyze genes at the overall level of the expression profile of OS related genes. The results showed that most of the upregulated genes were involved in immune system process, inflammatory response, regulation of nitric oxide biosynthetic process, positive regulation of reactive oxygen species biosynthetic process, regulation of reactive oxygen species metabolic process, and positive regulation of cytokine production, as shown in **Figure S2**.

### Protein-protein interaction network construction and enrichment analysis

**Figure 2:**
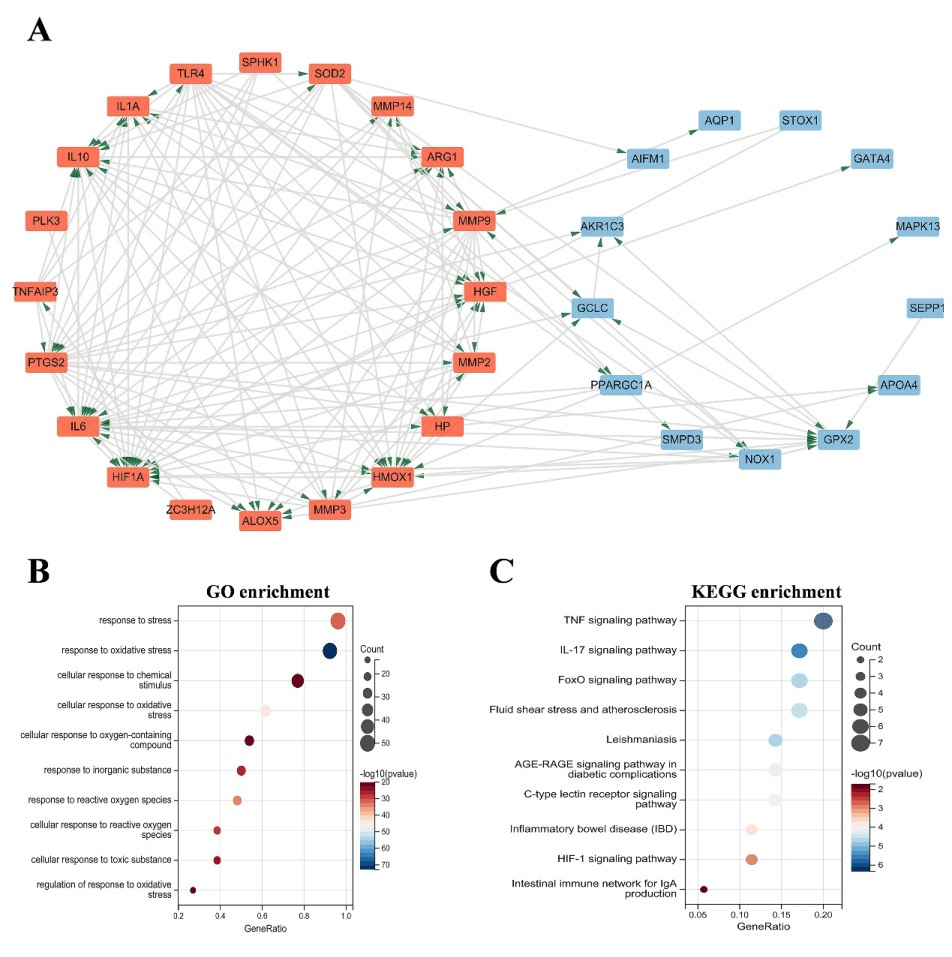
(A) PPI network diagram. Orange red indicates upregulated genes, and blue indicates downregulated genes. (B-C) GO and KEGG enrichment analyses of the modular genes. The size of the circle represents the number of genes involved, and the abscissa represents the frequency of the genes involved in the term total genes. PPI, protein-protein interaction; GO, Gene Ontology; KEGG, Kyoto Encyclopedia of Genes and Genomes.

The OS related DEGs were selected and input into the String database to construct a protein-protein interaction (PPI) network. Subsequently, Cytoscape software was employed to generate a visual representation of the PPI network **(Figure 2A-B)**, consisting of 35 nodes and 138 edges. GO analysis revealed that these genes are implicated in processes such as response to stress, response to oxidative stress, cellular response to chemical stimulus, cellular response to oxidative stress, cellular response to oxygen-containing compound, and response to reactive oxygen species **(Figure 2C)**. Additionally, KEGG analysis demonstrated that the DEGs were predominantly enriched in pathways such as the TNF signaling pathway, IL-17 signaling pathway, FOXO signaling pathway, inflammatory bowel disease (IBD), HIF-1 signaling pathway, and intestinal immune network for IgA production **(Figure 2D)**.

### Selection and analysis of hub genes

**Figure 3:**
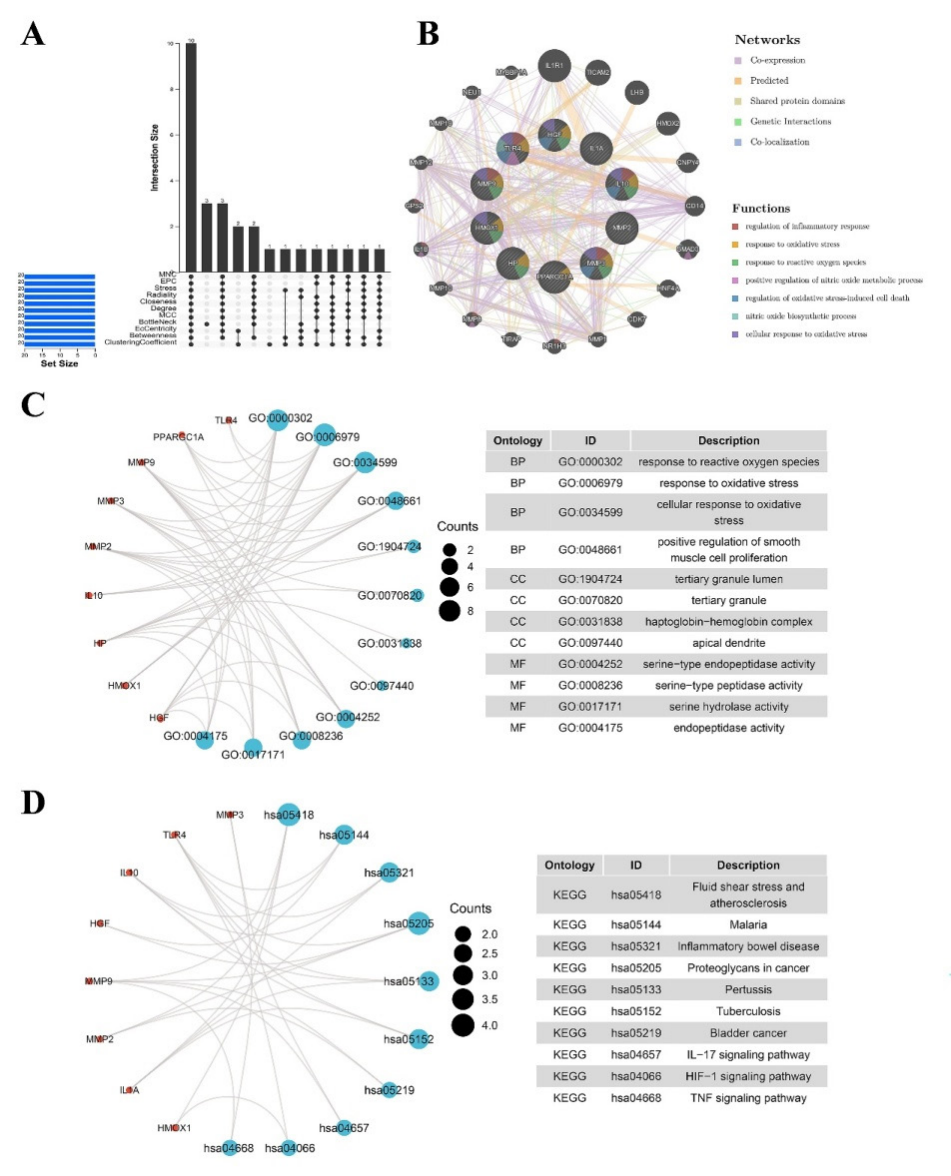
(A) The Venn diagram shows that seven algorithms have screened out nine overlapping hub genes. (B) Hub genes and their co-expression genes were analyzed via GeneMANIA. (C-D) GO and KEGG enrichment analyses of the hub genes. The outermost circle is term on the right, and the inner circle on the left represents the significant p-value of the corresponding pathway of the gene. GO, Gene Ontology; KEGG, Kyoto Encyclopedia of Genes and Genomes.

The identification of the top 20 hub genes was conducted through the utilization of seven algorithms provided by the cytoHubba plug-in. Upon intersecting the Venn diagrams, a set of ten shared hub genes emerged, comprising *MMP2*, *IL1A*, *MMP3*, *HGF*, *HP*, *IL10*, *PPARGC1A*, *TLR4*, *MMP9*, and *HMOX1* **(Figure 3A)**. The complete names of these genes, along with their respective functions, are detailed in **Table 1**. The GeneMANIA database was utilized to explore the co-expression network and functions associated with the identified hub genes. The investigation revealed a complex PPI network among these hub genes, characterized by co-expression at 81.82%, genetic interactions at 0.43%, co-localization at 0.15%, prediction at 13.02%, and shared protein domains at 4.58%** (Figure 3B)**. GO analysis showed that these genes are mainly enriched in response to reactive oxygen species, response to oxidative stress, cellular response to oxidative stress, positive regulation of smooth muscle cell proliferation **(Figure 3C)**. KEGG pathway analysis showed that they are mainly involved in the IL-17 signaling pathway, HIF-1 signaling pathway, TNF signaling pathway and IBD** (Figure 3D)**.

### Immune-infiltrating cell analysis

To understand the compositions of immune cells of intestinal tissue between NEC and normal groups, "CIBERSORT" method was used to discover the correlated immune cells involved in each bowel tissue 12. The enrichment fraction of immune-infiltrating cells was shown in **Figure S3A&B**. The results showed that B cells naïve, T cells CD8, Macrophages M2, Mast cells resting were significant inhibited in NEC tissue. Besides, Macrophages M0, Mast cells activated and Neutrophils were significantly activated NEC tissues, which suggested the potential regulatory roles of these immune cells in the progression of NEC. Furthermore, we conducted a correlation analysis on infiltrated immune cells, where the scores represent the extent of correlation** (Figure S3C)**. The correlation heatmap indicated that activated NK cells and resting mast cells showed the most synergistic effect (0.92), while resting mast cell and M2 macrophages, CD8 T cell and Neutrophils showed the most competitive effect (-0.88).

### Immune cell infiltration relationship and diagnostic value of hub genes in NEC

As indicated from the correlation analysis, we performed correlation analysis between Hub genes and immune cell infiltration. The results showed that *MMP9* displayed a significant positive correlation with M0 macrophages (r = 0.853, p = 0.003) and Neutrophils (r = 0.718, p = 0.029), and a significant negative correlation with Mast cells resting (r = -0.8, p = 0.01), CD8 T cells (r = -0.707, p = 0.033) **(Figure S4A)**. The association of the rest of Hub genes and immune cell infiltration were showed in **Figure S4B-J**. The ROC curve showed the probability of these hub genes as potential valuable biomarker in NEC **(Figure S6A-K)**. To enhance prediction performance, the integration of the ten hub genes was carried out to establish a multi-marker diagnostic model using logistic regression analysis. The ROC curve demonstrated that the multi-marker model exhibited effective predictive capability for the diagnosis of NEC, with an AUC value of 1.00 **(Figure S6L)**.

### Identification of candidate drugs for NEC

Ten hub genes were used to screen potential drugs for the treatment of NEC. According to the combined score, the top 10 candidate drugs were generated **(Table 2)**. Dexamethasone, the most enriched drug for nine hub genes. Dinoproston, Streptozotocin, PD 98059, Rosiglitazone, 9001-31-4, Glutathione, N-Acetyl-L-cysteine, Electrocorundum and Ac1l1wkq were identified as the potential drug targeting these OS hub genes. These potential small molecular compounds might serve as prevention or treatment for the occurrence and progression of NEC.

### Single-cell RNA-sequencing revealed the immune cellular heterogeneity between NEC and normal bowel tissue

**Figure 4:**
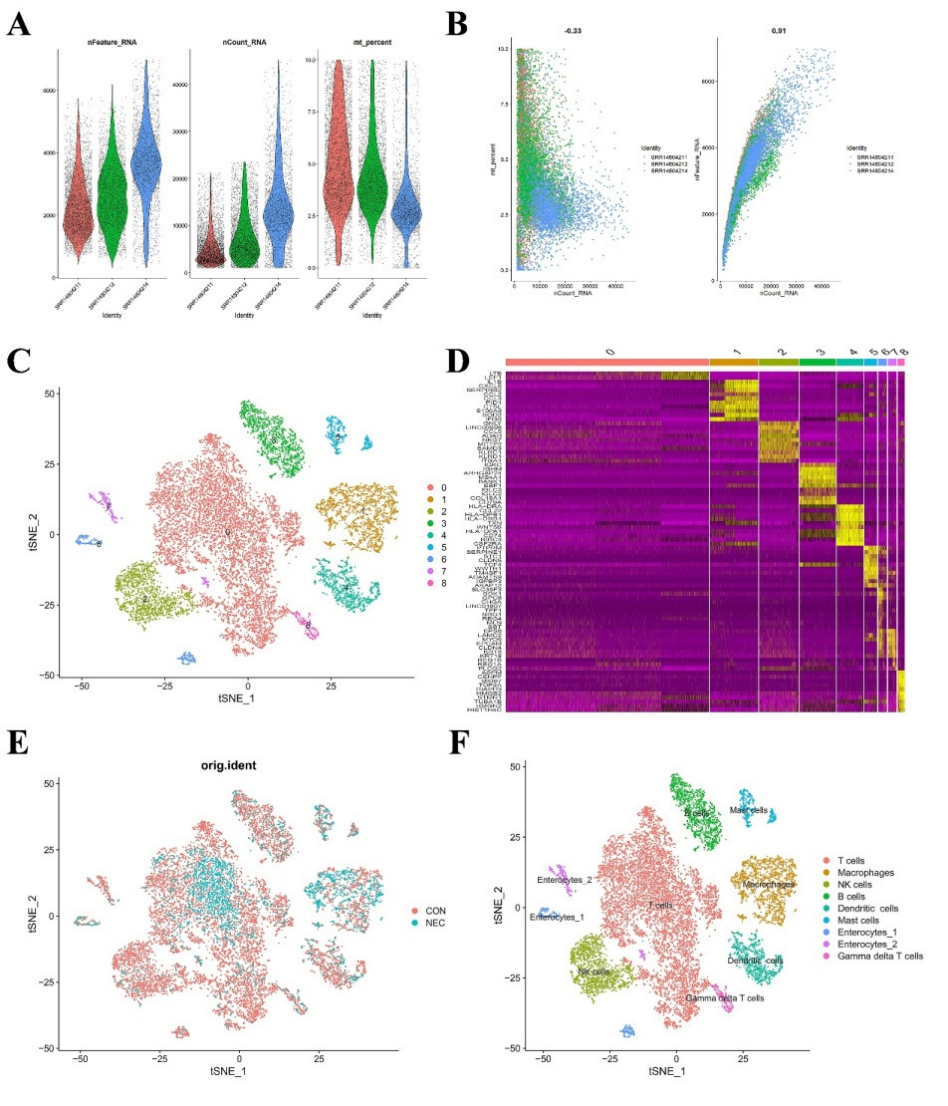
(A) Scatter plot after quality control and filtering of cells. (B) The left panel indicates that the mitochondria were the same with sequencing depth; the right panel suggests that the numbers of the detected genes were significantly related to the sequencing depth. (C) UMAP visualization of single cell RNA-sequencing data in NEC and CON, which identified 17 cell clusters. (D) The heatmap displays the top 5 genes of the identified markers genes of each cluster. (E-F) UMAP visualization of cell populations distribution between NEC and CON group.

In the preceding analysis, we observed aberrant distribution patterns of certain immune cells such as CD8 T cells, macrophages, and neutrophils in the NEC group. Consequently, we proceeded to construct a single-cell-level atlas depicting the heterogeneity of immune cells within NEC tissues, utilizing data from GSE178088. Following a meticulous quality control workflow aimed at excluding cells with low gene detection (<300 genes) and high mitochondrial gene content (>10%), a total of 13,695 cells expressing genes were retained for subsequent analysis **(Figure 4A)**. The count of detected genes exhibited a significant correlation with sequencing depth, and the mitochondrial unique molecular identifier (UMI) rate for each cell remained below 10% **(Figure 4B)**. Variance analysis was employed to identify the top 2,000 highly variable genes in each sample, and PCA was applied to determine relevant dimensions based on these principles. Following the removal of distinct samples using the Harmony integration algorithm, the top 50 PCs were computed based on the top 2,000 genes. Subsequently, the top 30 PCs with the lowest p-values were selected for subsequent dimensional reduction analysis. Utilizing an unsupervised dimensional reduction method through the tSNE algorithm, cell subtype clustering was conducted, resulting in the generation of 9 cell clusters. Marker gene detection for each cluster revealed significant heterogeneity among different cell populations **(Figure 4C-D)**. The specific types of cell clusters were identified using singleR, incorporating information from existing literature and databases. The heterogeneity among different cell clusters was characterized based on gene expression, resulting in the annotation of nine distinct subtypes of cells. Among these, seven were identified as various immune cell types, while the remaining subtypes were categorized as enterocytes **(Figure 4E-F)**.

### Hub genes distribution and oxidative stress levels among different cell populations

The distribution of the Hub genes at single cell level were showed in **Figure S6A**. The results displayed that the Hub genes were mainly expressed in immune cell, especially in Macrophages cell population. Utilizing the classified cell populations, we applied the "GSVA" algorithm to compute scores for OS related functions for each individual cell as shown in **Figure S6B**. The findings from single cell RNA-sequencing were in concordance with bulk RNA-sequencing, revealing that OS related functions in NEC tissues were notably elevated compared to those in CON tissues. We also assessed the OS related functions for each specific cell subtype. The results showed that the macrophages exhibited the highest OS related scores. Among immune cells, macrophages were shown as the strongest correlation immune cells with OS related functions. We next observed the OS related functions among different cell subtypes. The gene functional enrichment analysis was performed with the DEGs in the Macrophages cell population between NEC and CON group as shown in **Figure S6C**.

### Macrophage subtype populations in the development of NEC

**Figure 5:**
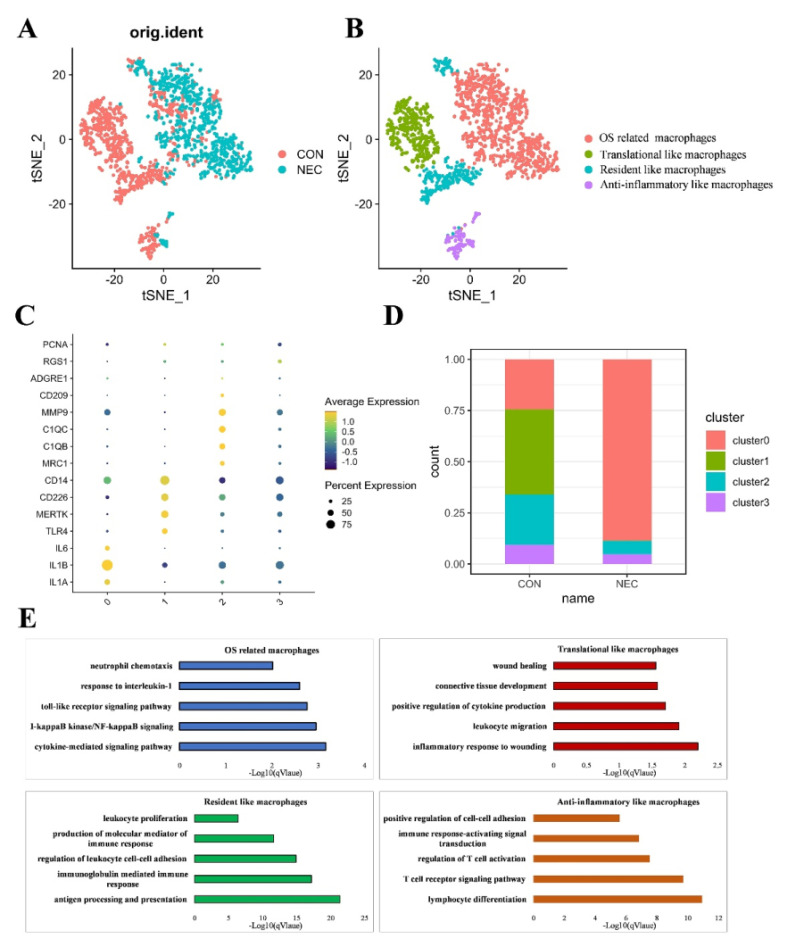
(A-B) UMAP visualization of cell subpopulations in macrophages in NEC and CON group. (C) Four different macrophage subtypes and their specific marker gene expression levels, with brightness indicating log-normalized average expression, and circle size representing the percent expressed. (D) The number cells of macrophage subtypes in NEC and CON group. (E) Bar plots showing the -log10 (p-value) from enrichment analysis of representative GO biological functions among different macrophage subtypes.

Within immune cells, macrophages exhibited the highest levels of OS related functions in NEC tissues. Subsequently, we conducted an analysis focusing on macrophage specific features that underwent dynamic changes during NEC. Consequently, we extracted and conducted a re-clustering analysis specifically for the macrophage population using the tSNE dimensional reduction method. In total, we analyzed 1,690 macrophage cells using variable genes and identified four distinct sub-clusters. Leveraging unique gene signatures, we characterized the following four macrophage subtypes, each holding distinct biological significance for downstream analysis: OS related macrophages (IL1A+EREG+macrophages), Translational like macrophages (CD14+ CD226+macrophages), Anti-inflammatory like macrophages (C1QB+ MMP9+macrophages) and Resident like macrophages (ITGAM^low^CD68^low^ monocytes/macrophages) **(Figures 5A, B)**. The normalized expression levels of representative marker genes for each subtype were shown in** Figure 5C**. The distribution of distinct cell populations between NEC and CON group is shown in **Figures 5D**. To comprehensively characterize the subtypes of macrophages, we identified DEGs for each subtype. The top 50 DEGs for each subtype were then subjected to GO biological functions analysis, as shown in **Figure 5E**. DEGs of OS related macrophages (the dominant population in NEC tissues) were enriched in functions like "acute inflammatory response," "ROS metabolic process," and "response to oxidative stress"; besides, they mainly expressed markers of M1 (*IL1A*, *IL1B* and *IL6*), which were considered to act roles like M1. Translational like macrophages had DEGs that were mainly associated with activated inflammatory response like "inflammatory response to wounding", "leukocyte migration" and "positive regulation of cytokine production". As for Anti-inflammatory like macrophages, they were mainly involved in functions of regulating effects, such as "lymphocyte differentiation", "T cell receptor signaling pathway" and "regulation of T cell activation". For Resident like macrophages, the DEGs were related to "antigen processing and presentation", "immunoglobulin mediated immune response" and "regulation of leukocyte cell-cell adhesion".

### Validation of hub genes expression in NEC mice model

**Figure 6:**
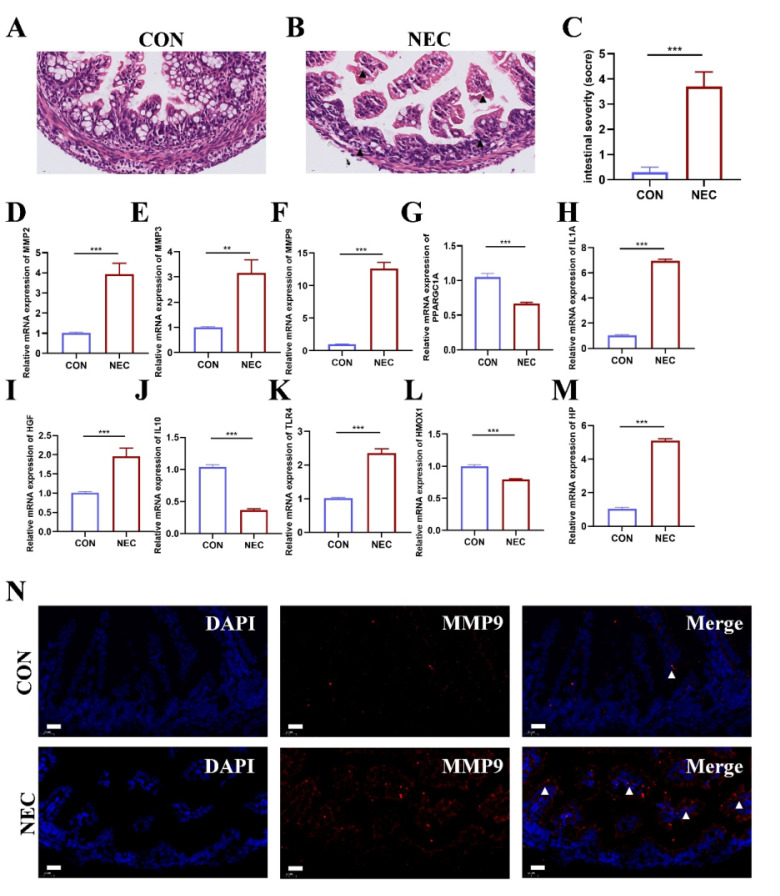
(A-B) Representative images of intestinal tissue sections in NEC and CON group stained by hematoxylin and eosin (n=5). Scale bar=20μm. (C) The pathological grade of the intestine tissue in NEC and CON group (n=5). *, p < 0.05; **, p < 0.01; ***, p < 0.001. (D-M) Validation of the ten hub genes' mRNA expression in intestinal tissue of NEC and CON group (n=3). *, p < 0.05; **, p < 0.01; ***, p < 0.001. (N) Representative immunofluorescent staining images of MMP9 expression in intestinal tissue of NEC and CON group (n=5).

NEC murine model was established to detect the mRNA expression of OS related Hub genes. Representative histological sections are shown in **Figure 6A-B**. The degree of histological damage in the NEC group was much greater than in the control group **(Figure 6C)**. RT-qPCR was performed to confirm the expression of the hub genes in NEC tissue. As shown in **Figure 6D-M**, we found that the mRNA expression of hub genes *MMP2*, *MMP3*, *MMP9*, *IL1A*, *HGF*, *TLR4*, and *HP* was significantly higher in the NEC group than control group. The expression of *PPARGC1A*, *IL10* and *HMOX1* was reduced in the NEC group compared to control group. Among these results, the expression of *MMP9* was more than tenfold higher than that of the control group. Therefore, we further examine the location of *MMP9* expression in intestinal tissues in both groups. As displayed in **Figure 6N**, in control group, *MMP9* is essentially unexpressed or sparsely expressed. In NEC murine model group, *MMP9* expressed abundantly in the intestinal mucosa.

## Discussion

Oxidative stress has been implicated as an important pathophysiological condition to contribute to NEC but not fully explained. In this study, we integrate the bulk RNA sequencing and Single cell RNA sequencing to depict the OS landscape in NEC for the first time. We identified an OS related subpopulation of macrophages in NEC and discovered OS related hub genes may be potential therapy targets of NEC.

Herein, we obtained 465 OS related genes, based on the differential analysis, a total of 53 OS related DEGs were filtered in NEC. GSEA results showed that the target proteins were mainly involved in immune system process, inflammation response, regulation of reactive oxygen species metabolic process, positive regulation of reactive oxygen species biosynthetic process and regulation of cytokine production. Besides, the GO/KEGG enrichment analysis of OS related DEGs showed that response to oxidative stress, cellular response to reactive oxygen and associated signaling pathways including TNF signaling pathway, IL17 signaling pathway, FOXO signaling pathway and HIF1A signaling pathway were significant enriched. Previous studies have demonstrated that the secretion of IL-17 by FOXP3+ lymphocytes lead to the disruption of tight junctions between adjacent enterocytes, facilitating bacterial translocation. Conversely, antibodies targeting IL-17 and inhibition of IL-17R have been shown to prevent intestinal injury in NEC 16. A finding supported by an earlier observation showing that the administration of anti-TNF agents can reduce NEC in mice 17. Our results suggest that in the development of NEC, OS related genes involve in immune regulation, inflammation, and cellular redox balance, imply an active role in oxidative stress responses. These findings highlight the significance of these proteins in orchestrating molecular events related to immune function, inflammation, and redox homeostasis.

Furthermore, we identified ten hub genes including *TLR4*, *IL1A*, *HGF*, *MMP9*, *HMOX1*, *HP*, *PPARGC1A*, *MMP3*, *MMP2* and *IL10*. *TLR4* has been extensively implicated in NEC. The induction of NEC involves the activation of *TLR4* on the intestinal epithelium by the microbiota in the premature host, resulting in enterocyte death, mucosal injury, and the translocation of bacteria into the circulation 16. *IL-1* is considered the prototypical pro-inflammatory cytokine and is induced in numerous cell types by various triggers. Active even at picogram concentrations, *IL-1* stimulates a wide range of inflammatory effects, including the production of other pro-inflammatory mediators, tissue damage, and the induction of fever. Studies on *IL1A* in NEC are rare. *IL1A* expression was rapid at 8 h and persisting for up to 34 h post-NEC induction 18. When compared to tissue resected from the same infants at re-anastomosis following recovery from NEC and non-inflamed tissue from healthy control infants, intestinal tissue resected from NEC infants showed elevated pro-inflammatory (*IL1B*, *IL1A*, *IL36A*, *IL36B*, *IL36G*) and reduced anti-inflammatory (*IL37*, *IL1R8*) mRNA expression of IL-1 family cytokines 19. IL10 functions as an anti-inflammatory cytokine, contributing to the maintenance of epithelial integrity and modulation of the mucosal immune system. Mice lacking IL10 (IL10^-/-^) exhibited more pronounced morphological and histological alterations compared to control mice. This was evident through heightened epithelial apoptosis, reduced localization of junctional adhesion molecule-1, and increased expression of inducible nitric oxide synthase in the intestinal tissues 20. Exogenous *IL10* administration reduced the mucosal damage. Hepatocyte growth factor (HGF) is a glycoprotein that plays a role in cellular survival, proliferation, and angiogenesis 21. Both BM and the fetal GI tract contain active *HGF*, which is involved in epithelial migration, proliferation, and tissue repair 22. *HGF* is vital for embryonic development as shown in a study where HGF deficient mice experience embryonic demise 23. Although the exact processes by which *HGF* works are not fully known, numerous animal models have demonstrated that HGF may both protect and heal intestinal tissue. Mice with deficient of HGF receptor, when subjected to DSS- or acetic acid-induced colitis, exhibited impaired colonic mucosal regeneration and higher mortality rates 24. Additionally, enteral HGF treatment reduced the frequency and intensity of NEC in rats 25. *HMOX1* plays a pivotal role in tissue protection, particularly under stress conditions. Its protective effects are crucially attributed to its antioxidative, antiapoptotic, and anti-inflammatory functions, which are mediated by the heme degradation products bilirubin and CO 26. Under inflammatory conditions, *HMOX1* can alter the proportions of Treg in the lamina propria of young mice, which may partially provide intestinal protection 27. The neonatal maturation of the pig intestine may depend on the functional development of glucose and lipid metabolisms, immune phagocyte differentiation, and inflammatory pathways. *PPARGC1A* may play a partial regulatory role in these processes 28. Matrix metalloproteinases (MMPs) have been demonstrated to play a role in mediating matrix remodeling and cell migration during tissue injury and repair in the intestine. Previous research indicates that several MMPs may contribute to tissue destruction and remodeling in NEC 29. Bister et al. proposed that the targeted inhibition of MMPs could serve as a novel therapeutic approach for managing intestinal inflammation associated with NEC 30. MMP3^-/-^ infected mice lack the immune response necessary to eradicate pathogen-causing bacteria from colons. This is most likely caused by the colon's decreased TNF-α expression and the delayed entry of CD4+ T cells into the lamina propria 31. Elevated blood serum concentrations of *MMP9*, *MMP2*, and *TIMP4* are associated with a progressive course of NEC with sepsis. Increases in the concentrations of MMP2>503 ng/ml, MMP9>812 ng/ml, TIMP4>1404 ng/ml can be regarded as statistically significant indicators of mortality of NEC. The suggested approach for determining the results of NEC in infants is distinguished by its excellent specificity (87%) and sensitivity (94%) 32. In this study, we identified that MMP9 with significant higher gene expression in NEC. Besides, Aberrant MMP9 expression was also observed in NEC intestinal tissue. Since *MMP9* has been widely studied as a key factor in the progression of IBD and is considered a potential therapeutic target. Numerous animal studies have demonstrated that knocking out *MMP9* or using anti-MMP9 antibodies can effectively alleviate colitis 33, 34. However, the specific roles of *MMP9* in the development of NEC have not been extensively investigated, and it might serve as a potential diagnostic marker or therapeutic target for NEC.

Furthermore, targeting this OS related hub genes, we identified some new small molecules targeting these hub genes for the treatment of NEC. In pups subjected to NEC-like damage, antenatal dexamethasone decreased systemic inflammation, maintained intestinal barrier integrity, and promoted SP-D expression on the intestinal mucosal surface 35. Dinoprostone, as known as peostaglandin E2, administration improved weight loss in pups after the onset of NEC and led to a healthier appearance, as well as promoted the survival of NEC pups 36. PD98059 is a potent and selective MEK inhibitor. Mice treated with PD98059 had less peritoneal exudation and polymorphonuclear cell migration brought on by zymosan. Additionally, PD98059 reduced the damage that zymosan caused to the liver, pancreas, and lungs as well as the increase in TNF-α and IL-1β plasma levels 37. Rosiglitazone is a clinical drug used for the treatment of type 2 diabetes, and it has the potential to inhibit the inflammatory response 38. By suppressing the NFRP3 inflammasome and TNF-α expression in macrophages, rosiglitazone could alleviate inflammation in radiation-induced intestinal damage 39.

Based on the bulk-RNA sequencing immune infiltration analysis, CD8 T cells, Macrophages, mast cells, and neutrophils were significant change in NEC. We further evaluated the OS score in the different cell populations in NEC. The results showed the OS score was significant higher in macrophages population, especially in NEC group.

During early life, intestinal macrophages, which are vital components of the innate immune system, exhibit functional plasticity and diversity in various aspects of intestinal development. These include resistance to pathogens, maintenance of the intestinal barrier, and regulation of the gut microbiota. Macrophages in preterm infants may play a role in the mechanisms leading to the occurrence of NEC. They are likely involved in maintaining intestinal barrier function, sensing changes in gut microbiota, and regulating the intestinal immune response. Maintaining the homeostasis of intestinal barrier function relies on the essential interaction between macrophages and intestinal epithelial cells 40. The breakdown of the neonatal intestinal barrier is a critical event in the initiation and progression of NEC 41.

Existing studies have shown that ROS are the basis for macrophages to kill invading microorganisms through NADPH oxidation-mediated bursts of oxidation. OS related macrophage is a kind of oxidative stress polarization state, which mainly occurs under oxidative stress. OS related macrophages perform their special functions by increasing the oxidoreductase system and alleviating oxidative stress. OS related macrophages have antioxidant and anti-inflammatory activities and can be involved in defending against oxidative stress and regulating inflammatory responses. We finally identified the four specific macrophages cell types at single cell level in the development of NEC.

There are some limitations in this study. Firstly, regarding the cross-sectional study design and the correlative nature of our findings, it is necessary for longitudinal and mechanistic studies to better understand causality. Secondly, for the candidate drugs and potential biomarkers identified in this study, more biological experiments are needed in the future study. It is indispensable for developing non-invasive diagnostic methods and the importance of longitudinal studies to establish causative relationships and validate biomarkers. Thirdly, considering the small sample size of NEC patients in the dataset, which limits the generalizability and statistical power of our results, future studies with larger sample sizes are essential.

Collectively, in this study, we identified OS related hub genes in NEC and integrated into diagnostic models and potential novel drugs were developed for NEC. Moreover, we identified the specific OS related macrophages subtype population in NEC. This study provided novel insights of the immune landscape and therapeutic strategies focused on oxidative stress in the development of NEC.

## Supplementary Material

Supplementary figures and tables.

## Figures and Tables

**Table 1:** The details of hub genes.

No.	Gene symbol	Full name	Function
1	MMP2	Matrix Metallopeptidase 2	This protein is thought to be involved in multiple pathways including roles in the nervous system, endometrial menstrual breakdown, regulation of vascularization, and metastasis.
2	IL1A	Interleukin 1 Alpha	This cytokine is a pleiotropic cytokine involved in various immune responses, inflammatory processes, and hematopoiesis. Produced by monocytes and macrophages, proteolytically processed and released in response to cell injury, and thus induces apoptosis.
3	MMP3	Matrix Metallopeptidase 3	This gene encodes an enzyme which degrades fibronectin, laminin, collagens III, IV, IX, and X, and cartilage proteoglycans. The enzyme is thought to be involved in wound repair, progression of atherosclerosis, and tumor initiation.
4	HGF	Hepatocyte Growth Factor	This gene encodes a protein that binds to the hepatocyte growth factor receptor to regulate cell growth, cell motility and morphogenesis in numerous cell and tissue types.
5	HP	Haptoglobin	This gene has also been linked to diabetic nephropathy, the incidence of coronary artery disease in type 1 diabetes, Crohn's disease, inflammatory disease behavior, primary sclerosing cholangitis, susceptibility to idiopathic Parkinson's disease.
6	IL10	Interleukin 10	This cytokine can block NF-kappa B activity, and is involved in the regulation of the JAK-STAT signaling pathway. Knockout studies in mice suggested the function of this cytokine as an essential immunoregulator in the intestinal tract.
7	PPARGC1A	PPARG Coactivator 1 Alpha	The protein encoded by this gene is a transcriptional coactivator that regulates the genes involved in energy metabolism. It provides a direct link between external physiological stimuli and the regulation of mitochondrial biogenesis, and is a major factor that regulates muscle fiber type determination.
8	TLR4	Toll Like Receptor 4	The protein encoded by this gene is a member of the Toll-like receptor family which plays a fundamental role in pathogen recognition and activation of innate immunity.
9	HMOX1	Heme Oxygenase 1	HMOX1 is a Protein Coding gene. Diseases associated with HMOX1 include Heme Oxygenase 1 Deficiency and Pulmonary Disease, Chronic Obstructive.
10	MMP9	Matrix Metallopeptidase 9	MMP9 is a Protein Coding gene, which plays an essential role in local proteolysis of the extracellular matrix and in leukocyte migration.

**Table 2:** Drug candidates targeting hub genes.

Term	Adjusted P-value	Combined Score	Genes
dexamethasone CTD 00005779	9.11E-12	13876.63425	IL10; MMP2; HGF; MMP3; HP; HMOX1; PPARGC1A; TLR4; MMP9
Dinoprostone BOSS	4.54E-10	6777.001927	IL10; IL1A; MMP2; MMP3; HMOX1; TLR4; MMP9
Streptozotocin CTD 00006786	4.54E-10	22894.81615	MMP2; MMP3; HMOX1; TLR4; MMP9
PD 98059 CTD 00003206	2.22E-09	4612.769207	IL10; IL1A; MMP2; HGF; MMP3; HMOX1; MMP9
Rosiglitazone BOSS	3.21E-09	6069.999978	IL10; MMP2; HMOX1; PPARGC1A; TLR4; MMP9
9001-31-4 BOSS	3.31E-09	5721.941068	IL10; MMP2; HP; HMOX1; TLR4; MMP9
Glutathione CTD 00006035	3.31E-09	5613.650149	IL10; MMP2; HMOX1; PPARGC1A; TLR4; MMP9
N-Acetyl-L-cysteine CTD 00005305	4.04E-09	3619.350935	IL10; MMP2; HGF; HMOX1; PPARGC1A; TLR4; MMP9
Electrocorundum CTD 00005364	8.19E-09	8228.248428	IL10; IL1A; MMP2; MMP3; MMP9
AC1L1WKQ CTD 00007050	8.31E-09	21941.19538	IL10; IL1A; MMP2; MMP9
