# Supplementary material for: Comprehensive Characterization of the Oxidative Stress Profiles in Neonatal Necrotizing Enterocolitis
Source: Int J Med Sci. 2025 Apr 9;22(9):2139–54. doi: 10.7150/ijms.109008 (PMC12035842; doi:10.7150/ijms.109008)
Supplement: Supplementary file 1 — Supplementary figures and tables. [file ijmsv22p2139s1.pdf]

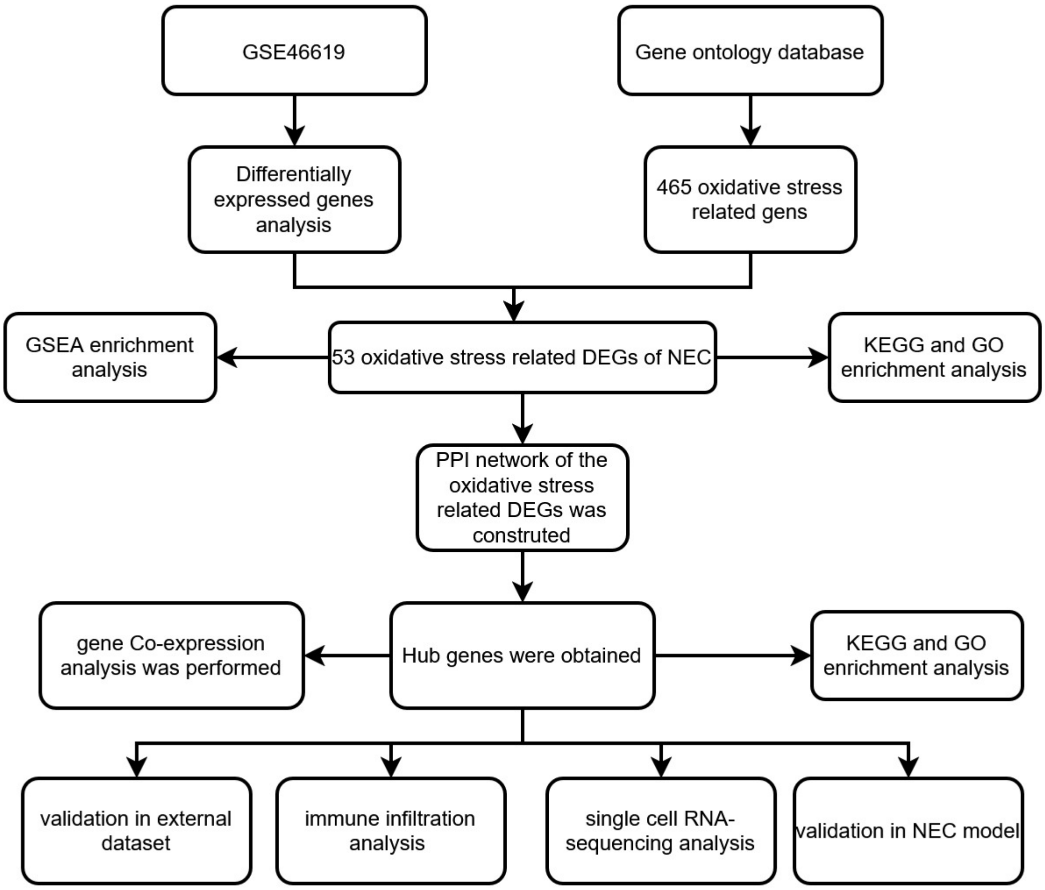

Figure S1. Flow chart of this study

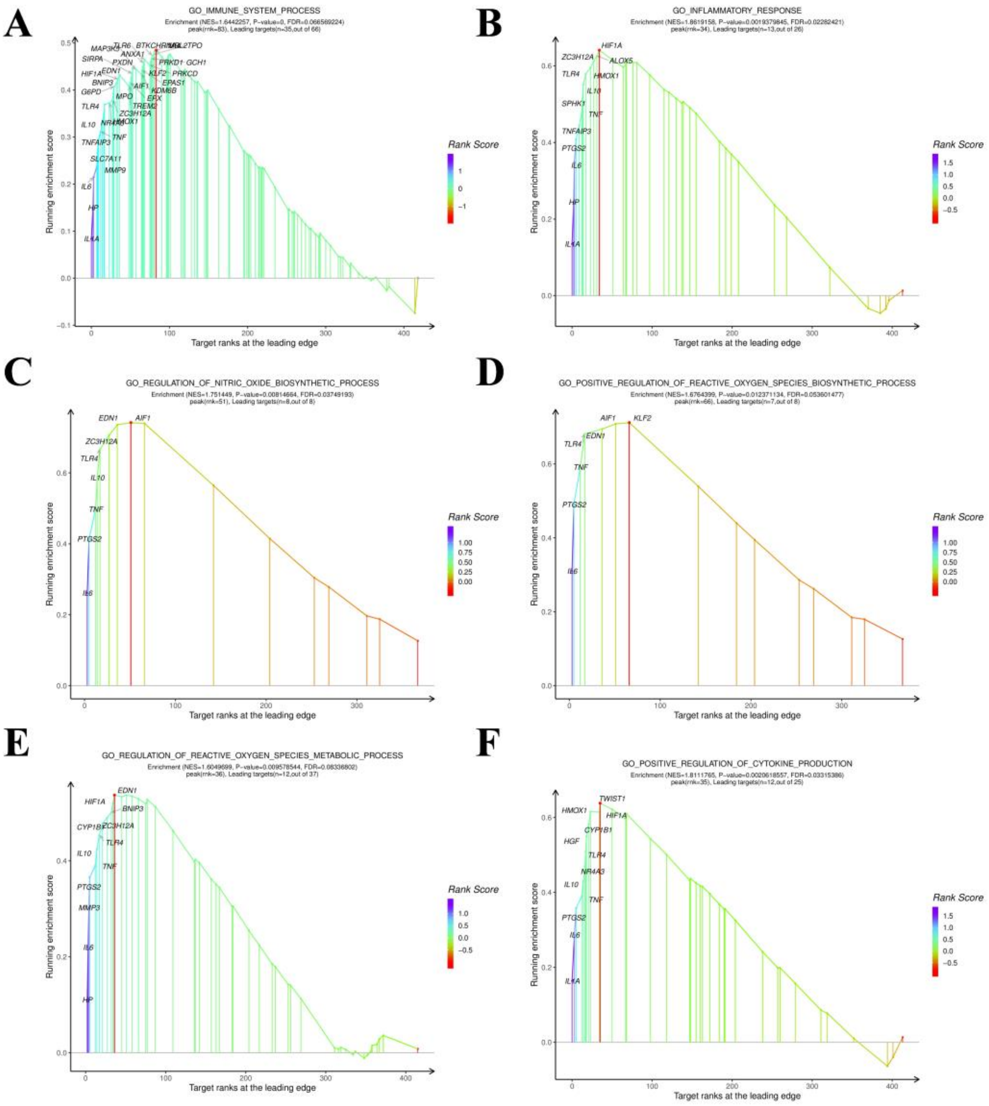

Figure S2. Gene set enrichment analysis of 488 OS-gene. FDR<0.25 and P value <0.05 were regarded as the cutoff criteria.

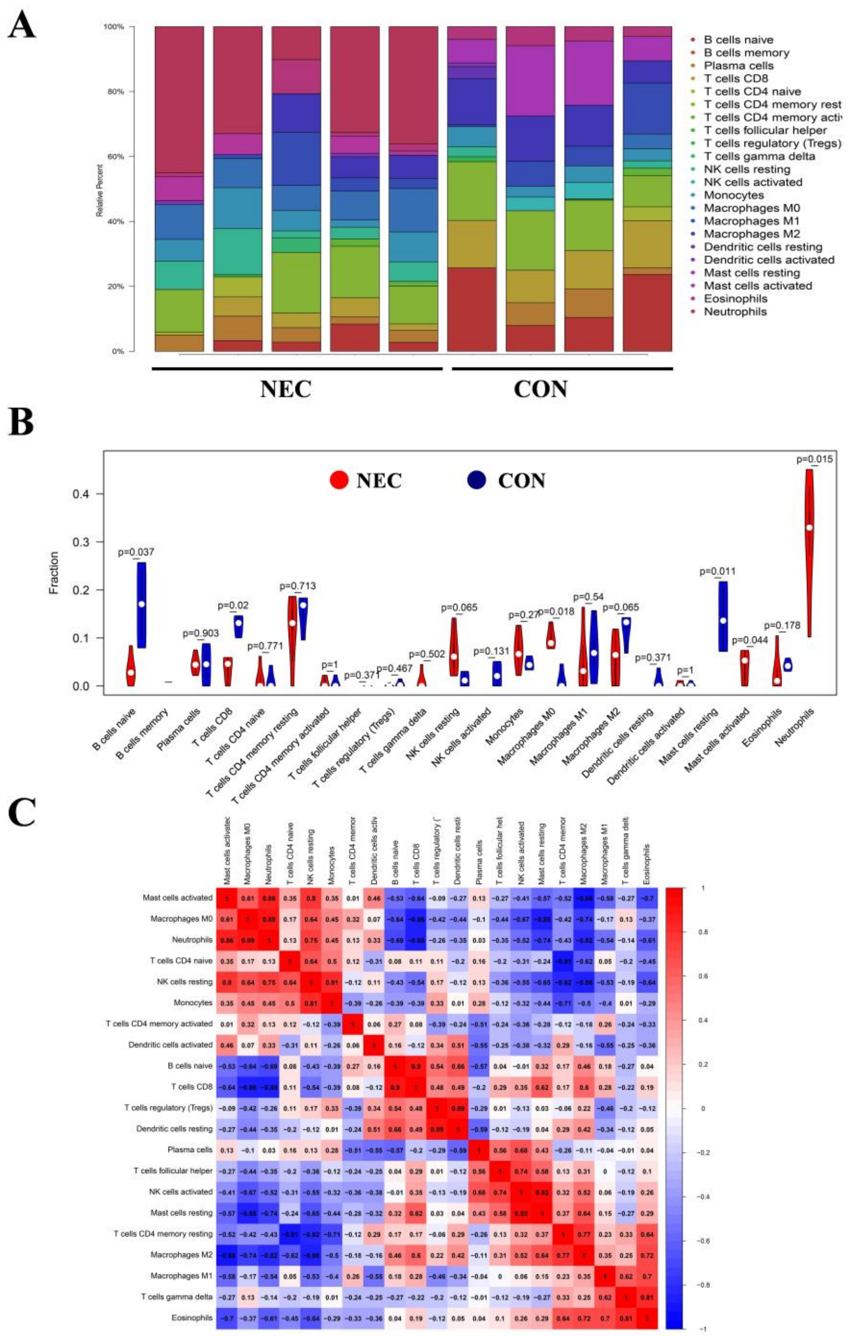

Figure S3. Immune cell infiltration patterns in NEC samples and CON samples. (A) Histogram of the proportions of 22 immune cell subpopulations in each NEC and non-CON sample. x-axis: GEO samples; y-axis: percentage of each immune cell type. (B) Violin plot showing the differentially infiltrated immune cells between the NEC and CON group. Blue represents the CON group and red represents the NEC group. (C) Correlation heatmap of all immune cells. Numbers in the small square represent Pearson's correlation coefficient between the two immune cells on the horizontal and vertical coordinates; red squares indicate positive correlation, and blue squares indicate negative correlation.

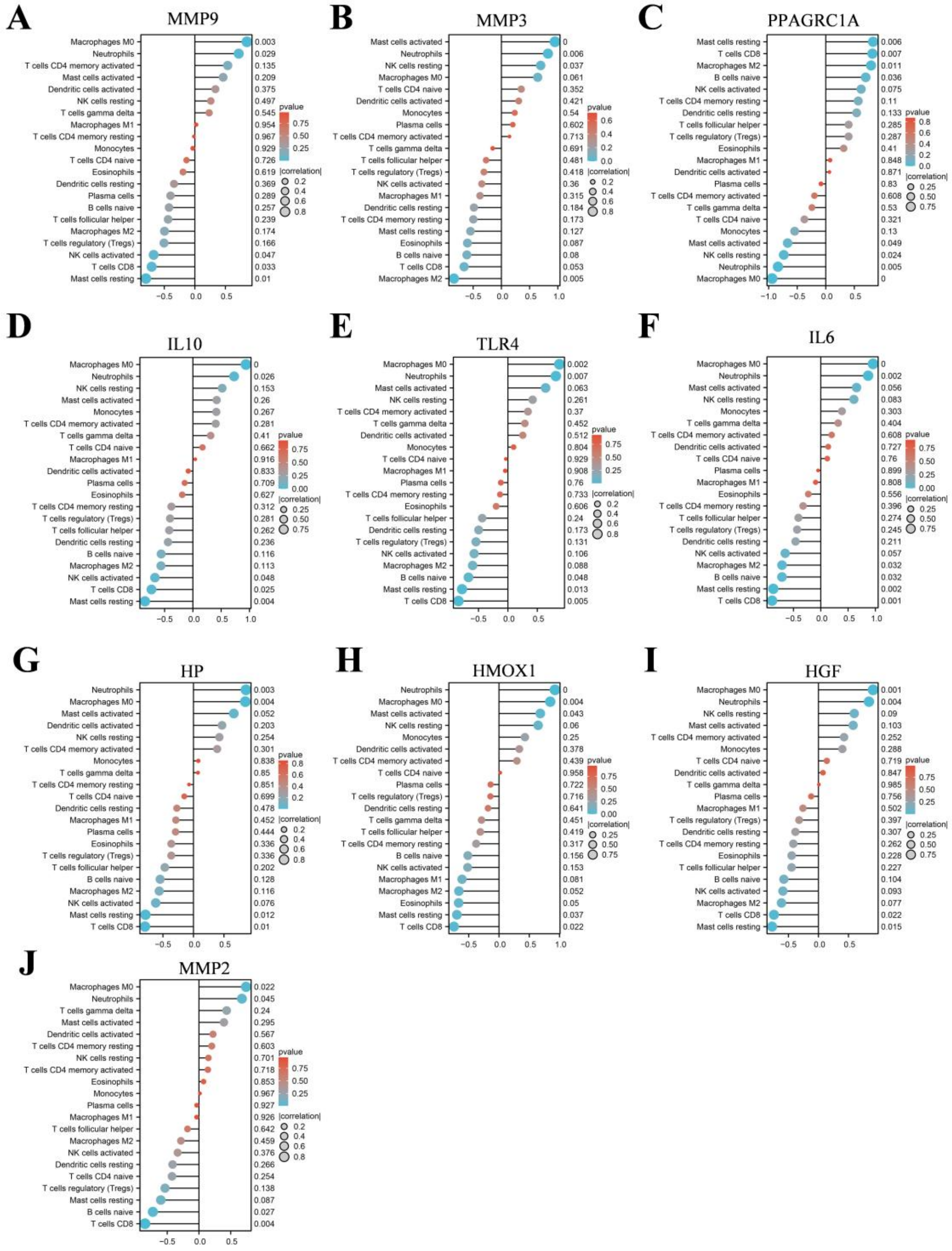

Figure S4. Correlation between the hub genes and infiltrating immune cells. The size of the dots represents the strength of the correlation between genes and immune cells, and the color of the dots represents the p-value. $p < 0.05$ was considered statistically significant.

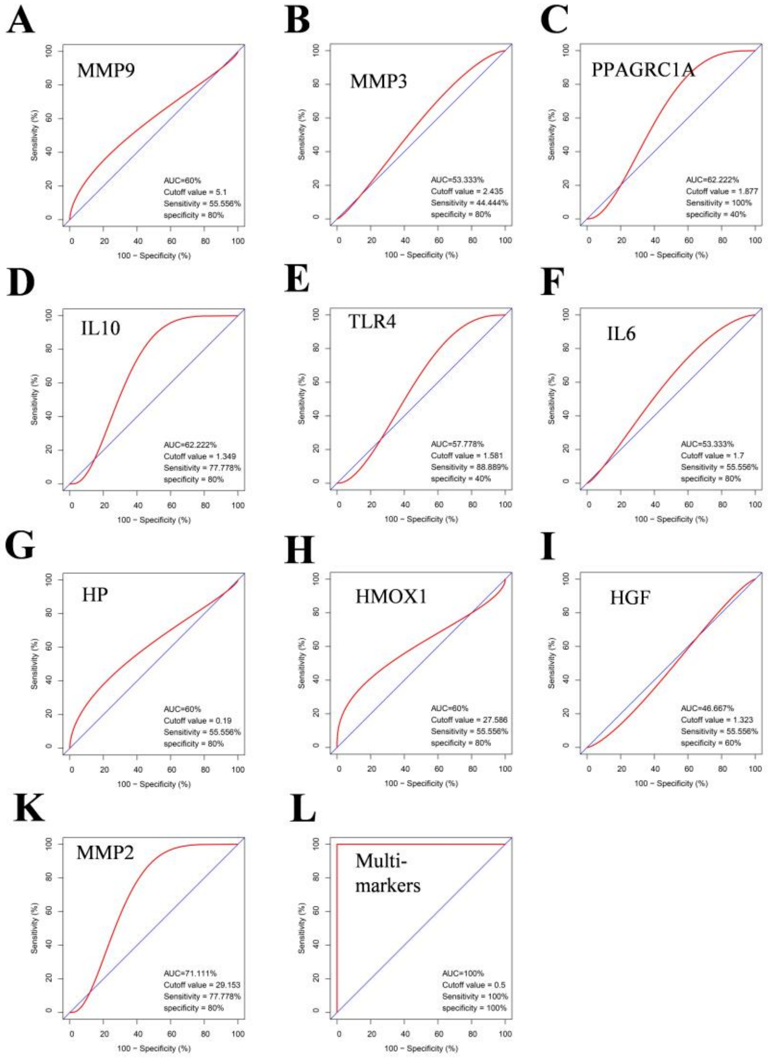

Figure S5. ROC curves for evaluating the accuracy of logistic regression analysis of the hub genes in dataset GSE64081.

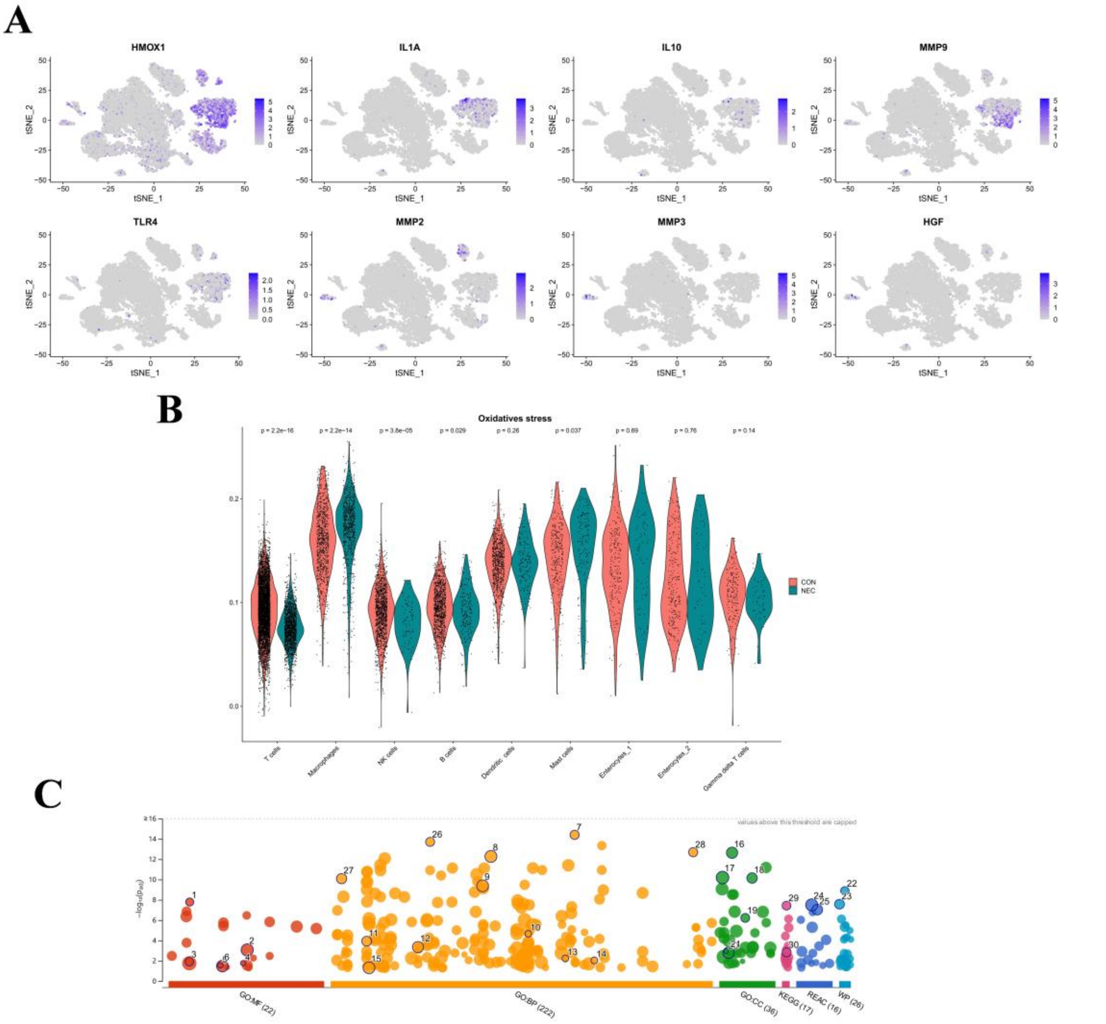

ID	Source	Term ...	Term Name	p_{adj} (query_1)
1	GO:MF	GO:0008009	chemokine activity	1.631×10^{-8}
2	GO:MF	GO:0042802	identical protein binding	8.515×10^{-4}
3	GO:MF	GO:0005518	collagen binding	1.217×10^{-2}
4	GO:MF	GO:0035662	Toll-like receptor 4 binding	1.704×10^{-2}
5	GO:MF	GO:0019863	IgE binding	2.836×10[illegible]
6	GO:MF	GO:0030234	enzyme regulator activity	3.305×10[illegible]
7	GO:BP	GO:0071621	granulocyte chemotaxis	3.951×10^{-15}
8	GO:BP	GO:0044419	biological process involved in interspecies interacti...	5.300×10^{-13}
9	GO:BP	GO:0042981	regulation of apoptotic process	4.124×10^{-10}
10	GO:BP	GO:0051238	sequestering of metal ion	2.198×10^{-5}
11	GO:BP	GO:0006909	phagocytosis	1.177×10^{-4}
12	GO:BP	GO:0019725	cellular homeostasis	4.479×10^{-4}
13	GO:BP	GO:0070486	leukocyte aggregation	5.783×10[illegible]
14	GO:BP	GO:0090280	positive regulation of calcium ion import	9.169×10[illegible]
15	GO:BP	GO:0007155	cell adhesion	4.875×10[illegible]
16	GO:CC	GO:0030141	secretory granule	2.289×10^{-13}
17	GO:CC	GO:0005615	extracellular space	6.356×10^{-11}
18	GO:CC	GO:0062023	collagen-containing extracellular matrix	7.075×10^{-11}
19	GO:CC	GO:0043202	lysosomal lumen	5.986×10^{-7}
20	GO:CC	GO:0008043	intracellular ferritin complex	1.121×10[illegible]
21	GO:CC	GO:0009986	cell surface	1.649×10[illegible]
22	WP	WP:WP3624	Lung fibrosis	1.286×10[illegible]
23	WP	WP:WP5115	Network map of SARS-CoV-2 signaling pathway	2.659×10[illegible]
24	REAC	REAC:R-HSA-16...	Immune System	3.139×10^{-8}
25	REAC	REAC:R-HSA-67...	Neutrophil degranulation	9.190×10^{-8}
26	GO:BP	GO:0030593	neutrophil chemotaxis	1.951×10^{-14}
27	GO:BP	GO:0002237	response to molecule of bacterial origin	8.172×10^{-11}
28	GO:BP	GO:1990266	neutrophil migration	2.029×10^{-13}
29	KEGG	KEGG:04657	IL-17 signaling pathway	3.680×10[illegible]
30	KEGG	KEGG:04668	TNF signaling pathway	1.476×10[illegible]

Figure S6. (A) Distribution of the eight hub genes (HMOX1, IL1A, IL10, MMP9, TLR4, MMP2, MMP3 and HGF) in NEC and CON group at single cell resolution. (B) The ROS score in each cluster in NEC and CON group at single cell level. (D) Functional enrichment analysis of the DEGs in the cluster of macrophages between NEC and CON group.

Supplementary table 1

**GSE64801**	**Type**	**source name**	**tissue**	**age**	**pathology**
GSM1580784	Control1	small intestine	ileum	33 5/7 weeks	Small intestinal perforation
GSM1580785	Control2	small intestine	ileum	26 6/7 weeks	Milk curd syndrome
GSM1580786	Control3	small intestine	ileum	26 5/7 weeks	Meconiumileus
GSM1580787	Control4	small intestine	ileum	33 6/7 weeks	Bowel obstruction
GSM1580788	Control5	small intestine	ileum	39 weeks	Small intestinal atresia
GSM1580775	NEC patient1	small intestine	ileum	32 weeks	acute preterm NEC
GSM1580776	NEC patient2	small intestine	ileum	25 3/7 weeks	acute preterm NEC
GSM1580777	NEC patient3	small intestine	ileum	26 2/7 weeks	acute preterm NEC
GSM1580778	NEC patient4	small intestine	ileum	27 2/7 weeks	acute preterm NEC
GSM1580779	NEC patient5	small intestine	ileum	24 6/7 weeks	acute preterm NEC
GSM1580780	NEC patient6	small intestine	ileum	26 2/7 weeks	acute preterm NEC
GSM1580781	NEC patient7	small intestine	ileum	25 4/7 weeks	acute preterm NEC
GSM1580782	NEC patient8	small intestine	ileum	29 6/7 weeks	acute preterm NEC
GSM1580783	NEC patient9	small intestine	ileum	29 2/7 weeks	acute preterm NEC
**GSE46619**	**Type**	**source name**	**tissue**	**stage**	**diagnosis**
GSM1133296	NEC1, BIOLOGICAL REPLICATE 1	necrotising enterocolitis (NEC)	bowel tissue	neonatal	necrotising enterocolitis (NEC)
GSM1133297	NEC2, BIOLOGICAL REPLICATE 2	necrotising enterocolitis (NEC)	bowel tissue	neonatal	necrotising enterocolitis (NEC)

GSM1133298	NEC3, BIOLOGICAL REPLICATE 3	necrotising enterocolitis (NEC)	bowel tissue	neonatal	necrotising enterocolitis (NEC)
GSM1133299	NEC4, BIOLOGICAL REPLICATE 4	necrotising enterocolitis (NEC)	bowel tissue	neonatal	necrotising enterocolitis (NEC)
GSM1133300	NEC5, BIOLOGICAL REPLICATE 5	necrotising enterocolitis (NEC)	bowel tissue	neonatal	necrotising enterocolitis (NEC)
GSM1133306	Surg-CTL1, BIOLOGICAL REPLICATE 1	normal bowel	bowel tissue	neonatal	normal bowel
GSM1133307	Surg-CTL2, BIOLOGICAL REPLICATE 2	normal bowel	bowel tissue	neonatal	normal bowel
GSM1133308	Surg-CTL3, BIOLOGICAL REPLICATE 3	normal bowel	bowel tissue	neonatal	normal bowel
GSM1133309	Surg-CTL4, BIOLOGICAL REPLICATE 4	normal bowel	bowel tissue	neonatal	normal bowel
**GSE178088**	**Type**	**source name**	**sex**	**tissue**	**age**
GSM5379914	1102_Fetal	Fetal small intestine	female	Fetal small intestine	21 weeks
GSM5379913	1100A_Fetal	Fetal small intestine	not determined	Fetal small intestine	21 weeks
GSM5379915	1127Neontal	Neonatal small intestine	female	Neonatal small intestine	37 weeks
GSM5379916	1164Neonatal	Neonatal small intestine	female	Neonatal small intestine	37 weeks
GSM5379917	1074NEC	Preterm small intestine		Preterm small intestine	31 weeks
GSM5379918	1109NEC	Preterm small intestine	female	Preterm small intestine	39 weeks

Supplementary table 2

gene
OSER1
OSGIN1
OSGIN2
ALKBH2
TLDC2
OXR1
GPX5
URS00004636A3_9606
MEAK7
PYCR1
LANCL1
GPX7
SRXN1
GPX8
PYROXD1
GPX6
NCOA7
ALKBH3
ZNF622
NOX1
VNN1
ANKZF1
ERMP1
TRAP1
IPCEF1
GPX2
TBC1D24
MSRA
CCS
PYCR2
CRYGD
ZNF277
VKORC1L1
cat_human
NONO
MCL1
MELK
GSKIP
PDK1
PRODH
DIABLO

ENDOG
MMP3
BAG5
PAGE4
ANKRD2
SLC7A11
CHD6
GPR37
GPR37L1
PJVK
SIGMAR1
URS0000CCE0E6_9606
SLC25A24
MAPK7
DHRS2
MYEF2
MGST1
VRK2
SOD3
ERCC6L2
MPV17
GSR
IL18BP
AGAP3
NME8
RBM11
WNT16
URS0000324096_9606
SELENON
STK24
PRDX1
PPIA
SCARA3
MSRB3
PON2
C19orf12
GPX3
CA3
ATOX1
STK26
FBLN5
ARL6IP5
CHCHD2

RAD52
SLC4A11
TMEM161A
HTRA2
TRPM2
PLA2R1
GGT7
ACOX2
ALKBH1
SOD2
NME5
GFER
GPX1
PINK1
STK25
HNRNPM
RWDD1
BRF2
ADNP2
MAP1LC3A
ADPRS
STX2
FANCC
ALDH3B1
MGAT3
SUMO4
NUDT2
NET1
FBXW7
ALKBH5
UCP3
RNF112
UBQLN1
MAPK13
AIFM2
PPIF
UCP1
TXN
PDE8A
PAWR
MAP3K5
SFPQ
PXN

ATF4
P4HB
RIPK1
SOD1
PARK7
NOL3
TLR6
HSPB1
NFE2L2
MCTP1
MGMT
CYB5B
MSRB2
ATRN
PSMB5
ANGPTL7
TPO
SELENOP
PXDNL
DGKK
FBXO7
FZD1
FTO
REST
PDCD10
FANCD2
STAU1
TRPA1
HDAC6
MET
APTX
MICB
RACK1
URS0000028BB8_9606
URS0000812128_9606
URS00004E5112_9606
IMPACT
EIF2S1
MT-CO1
URS00003768C5_9606
ERCC8
SLC25A23
NFE2L1

PDK2
ETV5
GJB2
FABP1
TP53INP1
PRKRA
GLRX2
LONP1
KDM6B
ROMO1
MPO
PRR5L
KEAP1
PRDX5
PCGF2
CPEB2
PLEKHA1
EPAS1
NR4A3
PPP1R15B
DNAJC15
SESN2
WNT1
TSC1
FUT8
PNPLA8
MAPKAP1
SESN3
SESN1
THG1L
TXN2
GSS
ETFDH
NAPRT
LPO
HBA1
TXNRD2
SGK2
TAT
HIF1A
PRKN
URS00002075FA_9606
URS00005B3525_9606

PARP1
KLF2
MT-RNR2
FXN
G6PD
URS000006FDD4_9606
URS00006054DA_9606
FOXO3
TPM1
ATP2A2
URS000030BD69_9606
FOXP1
HGF
DDR2
GPX4
BANF1
NUDT1
NUDT15
TLR4
OXSR1
ARNT
SELENOS
ALOX5
MMP2
AIFM1
PENK
MAPK9
SIRPA
NCF1
ERN1
ARNTL
URS00004C9052_9606
NDUFB4
NDUFS8
NDUFA6
NDUFS2
NDUFA12
MT-ND6
MT-ND5
MT-ND3
IL10
ERO1A
PRDX2

DHFR
STAU2
ABCC1
GCH1
FYN
COA8
PTGS1
PXDN
CYGB
PSIP1
HAO1
LIAS
HP
RCAN1
EPX
HMOX2
URS0000476BE1_9606
URS00005743AE_9606
NQO1
ZNF580
PPARGC1B
CAMKK2
PDGFD
CHUK
CAT
PTPRK
DAPK1
RHOB
ECT2
SETX
PNPT1
PRDX3
PDLIM1
PPP2CB
STC2
GATA4
CYP1B1
STOX1
SIRT1
INS
URS0000338542_9606
SIN3A
URS000024463E_9606

URS000055128B_9606
FOXO1
ABCD1
PRKAA2
FOS
PCNA
MAPK8
AKT1
LRRK2
HMOX1
PRDX4
MBL2
NEIL1
RBPMS
ERCC3
CHRNA4
KRT1
RRM2B
DUOX2
DUOX1
MMP9
NR4A2
SPHK1
MAPK3
JUN
MAPK1
ABL1
PML
UCP2
SHPK
SELENOK
AKR1C3
APEX1
RIPK3
NUPR1
ATP13A2
APOA4
STX4
SMPD3
BNIP3
PPARGC1A
CD36
PRKCD

AQP1
TNF
TREM2
AK4
S100A7
SLC1A1
SLC23A2
MTF1
TXNIP
IDH1
DHCR24
HBB
SDHD
CYP2E1
SDC1
PRDX6
TDG
MAPT
OGG1
PNKP
CRYAB
XRCC1
CRK
GSTP1
RELA
MT3
NOS3
EZH2
RPS3
ERCC6
URS000039ED8D_9606
BID
RGS14
FOSL1
TOR1A
PDGFRA
BTK
TREX1
TNFAIP3
ARG1
ATP7A
ERCC1
CTNNB1

PDE12
ZC3H12A
HSPA1A
BECN1
FER
PRKD1
STAT6
HSPA1B
CDK1
HDAC2
AXL
EDNRA
AIF1
TP53
MT-ATP6
MT-CO2
MT-ND4
BAK1
BAD
IL1A
PTGS2
GCLM
ALS2
CAPN2
STOML2
BCL2
ERCC2
SCGB1A1
SP1
APOD
WRN
TACR1
abeta-42-oligomer_human
PKD2
SIRT2
EGFR
HSF1
ATF2
PLK3
CASP3
SNCA
AREG

ALAD
ADAM9
PTPRN
ABCB11
JAK2
HYAL1
PRKAA1
BMP7
VCP
ANXA1
GCLC
PAX2
TWIST1
ADIPOQ
NDUFC2
SHMT2
FKBP1B
SLC8A1
UCN
UBE3A
MMP14
MACROH2A1
COL1A1
KCNA5
CD38
PTK2B
SRC
STAT1
HYAL2
EDN1
IL6
RHOA
PRNP
MSH2
APP
PDGFRB
PSEN1
APOE
